# Effects of MMP-9 inhibition by doxycycline on proteome of lungs in high tidal volume mechanical ventilation-induced acute lung injury

**DOI:** 10.1186/1477-5956-8-3

**Published:** 2010-01-29

**Authors:** Adrian Doroszko, Thomas S Hurst, Dorota Polewicz, Jolanta Sawicka, Justyna Fert-Bober, David H Johnson, Grzegorz Sawicki

**Affiliations:** 1 Department of Pharmacology, University of Saskatchewan, Saskatoon, Saskatchewan, Canada; 2 Department of Medicine, University of Saskatchewan, Saskatoon, Saskatchewan, Canada; 3 Department of Medicine, University of Alberta, Edmonton, Alberta, Canada; 4 Department of Clinical Chemistry, Medical University of Wroclaw, Wroclaw, Poland; 5 On leave of absence from Department of Internal Medicine and Hypertension, Wroclaw Medical University, Wroclaw, Poland

## Abstract

**Background:**

Although mechanical ventilation (MV) is a major supportive therapy for patients with acute respiratory distress syndrome, it may result in side effects including lung injury. In this study we hypothesize that MMP-9 inhibition by doxycycline might reduce MV-related lung damage. Using a proteomic approach we identified the pulmonary proteins altered in high volume ventilation-induced lung injury (VILI). Forty Wistar rats were randomized to an orally pretreated with doxycycline group (n = 20) or to a placebo group (n = 20) each of which was followed by instrumentation prior to either low or high tidal volume mechanical ventilation. Afterwards, animals were euthanized and lungs were harvested for subsequent analyses.

**Results:**

Mechanical function and gas exchange parameters improved following treatment with doxycycline in the high volume ventilated group as compared to the placebo group. Nine pulmonary proteins have shown significant changes between the two biochemically analysed (high volume ventilated) groups. Treatment with doxycycline resulted in a decrease of pulmonary MMP-9 activity as well as in an increase in the levels of soluble receptor for advanced glycation endproduct, apoliporotein A-I, peroxiredoxin II, four molecular forms of albumin and two unnamed proteins. Using the pharmacoproteomic approach we have shown that treatment with doxycycline leads to an increase in levels of several proteins, which could potentially be part of a defense mechanism.

**Conclusion:**

Administration of doxycycline might be a significant supportive therapeutic strategy in prevention of VILI.

## Background

Acute lung injury (ALI) and its more severe form, acute respiratory distress syndrome (ARDS), are characterized by an acute inflammation and disruption of the alveolar-capillary membranes leading to alveolar flooding with protein-rich edema fluid. Mortality rates resulting from ALI and ARDS range from 18 to 54.7% [1]. Although mechanical ventilation (MV) is an important supportive strategy for patients with ARDS, it is also known to further lung injury in certain conditions of mechanical stress, leading to ventilation-induced lung injury (VILI). The mechanisms by which conventional MV exacerbates lung injury and inflammation are of considerable clinical significance. The potential importance of VILI in the clinical treatment of critically ill patients has been well established by recent clinical trials [2,3], where a relative risk reduction of 22% in patients ventilated with the lower tidal volume has been shown. The results from these studies indicate that mortality attributable to VILI is at least 9 to 10% in such patients. Therefore, despite the significant progress that has been recently made in emergency medicine, further research concerning the pathophysiology of ALI is needed in order to indicate new therapeutic targets preventing pulmonary damage.

Several experimental and clinical studies led to the hypothesis that the deleterious effects of MV might be mediated by local inflammation and the systemic release of inflammatory cytokines (biotrauma) [4-6]. During ALI/ARDS, various proinflammatory cytokines and chemokines are up-regulated and contribute to the initiation and propagation of the inflammatory response. In clinical studies, changes in levels of biological markers have been used primarily in an effort to identify VILI rather than to study disease pathogenesis. The ventilator-associated changes in levels of some biological markers have been correlated with patient outcomes, including duration of MV, organ failures, length of hospital stay, and mortality [7-9]. Despite various pharmacologic interventions developed in the last two decades that aim at specific targets, such as cytokines and adhesion molecules, the therapeutic strategy for this syndrome remains to be established. Although alveolar epithelial injury is a major determinant of outcome in patients with ALI, there is still no reliable biological marker of alveolar epithelial injury.

Recently, new mechanisms in the pathology of acute respiratory failure have shifted the focus to lung mechanics, tissue damage, remodeling, and the systemic effects derived from the mechanical stress imposed by the ventilator in patients with ARDS [7]. The conversion of physical signals such as contractile forces or external mechanical perturbations into chemical signaling events is a fundamental cellular process that occurs at the cell-extracellular matrix contact, known as focal adhesions, thus modifying cell viscoelastic properties, which may compromise the balance of forces in the alveolar epithelium [10]. Stretch-induced cell stiffening could compromise the balance of forces at the cell-cell and cell-matrix adhesions [4]. Mascarenhas et al. [11] in an *in vitro *study showed that *de novo *synthesis of hyaluronate in the extracellular matrix increases proinflammatory cytokines in VILI. Moreover, Taylor et al. [12] demonstrated that hyaluronate released after lung injury can stimulate endothelial cells to produce cytokines by activation of a Toll-like receptor 4-dependent mechanism, thus suggesting that endogenous components of the extracellular matrix can stimulate endothelial cells to trigger recognition of injury in the initial stages of the wound defense and repair response. Recent data from studies performed on pulmonary microvascular endothelial cells and isolated perfused rat lungs confirm the role of endothelial responses to stretch-inducing VILI [13]. High tidal volume ventilation (HVV) may induce focal adhesion formation and recruit leukocytes on the endothelial cells [14]. Haseneen and colleagues [13] demonstrated the existence of a connection between stretched endothelial cells and lung remodeling by release of matrix metalloproteinases (MMPs) activated through a membrane type-1 MMP (MT1-MMP) mechanism. The first evidence suggesting that MMPs are important in regulation of endothelial permeability was revealed by Soccal and co-workers using a lung ischemia/reperfusion (I/R) model [15]. Furthermore, it has been shown that inhibition of MMP-2 and MMP-9 protects the blood-brain barrier during cerebral ischemia and that there is an increase in MMP-2 activity resulting from an increase in oxidative stress [16]. In ALI, and mainly in ARDS, increased levels of MMP-2 and MMP-9 in the bronchoalveolar lavage (BAL) have been suggested to play a role in basement membrane disruption [17]. Studies in various lung injury models show that MMPs are strongly related to the pathogenesis of lung injury [18-20] and that MMP inhibitors (MMPI) decrease the extent of lung injury [21,22]. Therefore, understanding the extracellular as well as intracellular actions of MMPs might be crucial for studying molecular mechanisms of ALI, including those related to MV. The detrimental role of increased MMP-9 activity in pathogenesis of VILI has been widely explored [21,23-25] and there is also some evidence regarding the beneficial role of doxycycline in other models of ALI [26,27]. However, it has not been clearly established whether doxycycline, by inhibition of MMP-9, might play a beneficial role in limitation of VILI.

A better understanding of pathological mechanisms of ALI should help not only to alleviate the side effects of mechanical forces, but also to develop new therapeutic strategies. Moreover, it is necessary to develop potential pharmacologic targets to modulate the molecular and cellular effects of lung stretch to minimize the iatrogenic consequences of MV.

In the present study, using the pharmacoproteomic approach, we investigated the hypothesis that increased MMP-9 activity, involved in VILI, results in changes in pulmonary proteome, which could be minimized by MMP-9 inhibition by use of doxycycline.

Examination of global changes in protein levels due to VILI should provide new insights into the mechanisms involved in lung damage. Furthermore, it should lead to the development of novel therapeutic strategies as well as in establishment of more precise and sensitive diagnostic markers.

## Results

### Effect of ventilation on mechanical function of lungs and on gas exchange

**Figure 1:**
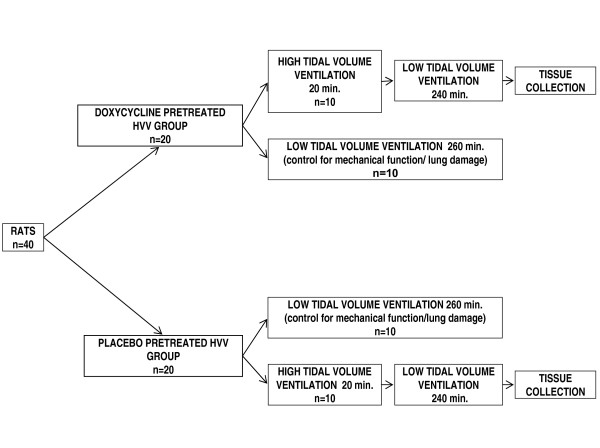
**Experimental protocol**. Forty Wistar rats were randomized to an orally pretreated with doxycycline group (n = 20) or to a placebo group (n = 20) each of which was followed by instrumentation prior to either low or high tidal volume mechanical ventilation. Afterwards, animals were euthanized and lungs were harvested for subsequent analyses.

Since treatment with doxycycline had no effect on either mechanical function of lungs or on parameters of gas exchange in low tidal volume ventilated lungs which were present in high tidal volume ventilated (HVV) lungs, we decided to perform biochemical analyses of lungs from only HVV groups (Figure 1). We postulated that HVV should result in changes similar in nature but greater in magnitude as compared to those observed in lungs ventilated with low tidal volume and therefore the observed changes in lung function should be much more pronounced.

**Figure 2:**
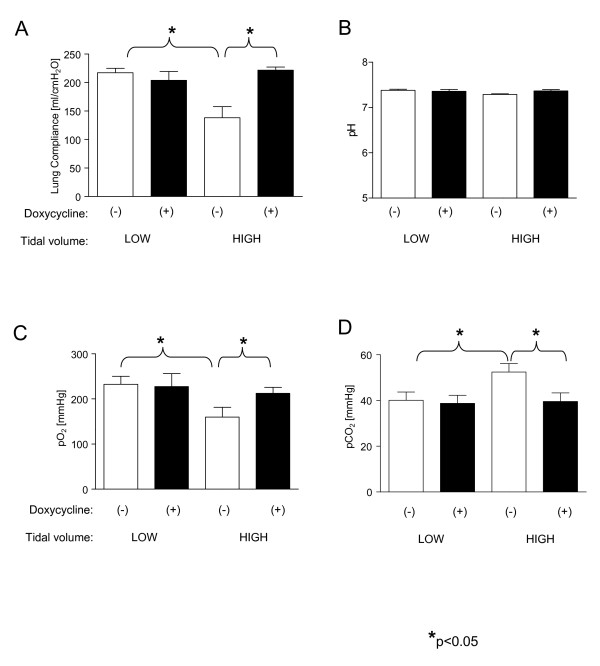
**Changes in lung function dependent on ventilation volume as well as on treatment with doxycycline**. A. Lung compliance in four subgroups separated by volume of ventilation and by treatment with doxycycline or placebo. *p < 0.05 for indicated pairs respectively. B. pH values in four subgroups separated by volume of ventilation and by treatment with doxycycline or placebo. C. Partial pressure of O_2 _in arterial blood at the end of mechanical ventilation in four subgroups separated by volume of ventilation and by treatment with doxycycline or placebo. *p < 0.05 for indicated pairs respectively. D. Partial pressure of CO_2 _in arterial blood at the end of mechanical ventilation in four subgroups separated by volume of ventilation and by treatment with doxycycline or placebo. *p < 0.05 for indicated pairs respectively.

Treatment with doxycycline resulted in a significant increase in lung compliance as compared to the placebo HVV treated group (221.8 ± 5.4 ml/cm H_2_O *vs*. 148.3 ± 5.4 ml/cm H_2_O in the placebo group, respectively, p = 0.028) which was comparable to levels observed in the low volume ventilation groups (Figure 2A). The values of blood pH in all subgroups remained at physiological levels which is shown in Figure 2B.

High tidal volume ventilation following placebo treatment resulted in significant worsening of gas exchange as assessed by a decrease in PaO_2 _(159.6 ± 21.9 mmHg *vs. *232 ± 18.1 mmHg in low ventilated placebo group, respectively, p = 0.027) as well as an increase in PaCO_2 _(52.4 ± 3.7 mmHg *vs. *40.1 ± 3.6 mmHg in low ventilated placebo group, respectively, p = 0.048). An impairment in gas exchange was reduced by pretreatment with doxycycline (PaO_2 _= 212.6 ± 13.3 mmHg, p = 0.048 vs. PaO_2 _in placebo treated HVV group and PaCO_2 _= 39.6 ± 3.7 mmHg vs. PaCO_2 _in placebo treated HVV group, respectively) (Figure 2C and 2D).

### Analysis and identification of proteins in lung homogenates separated by 2-DE

**Figure 3:**
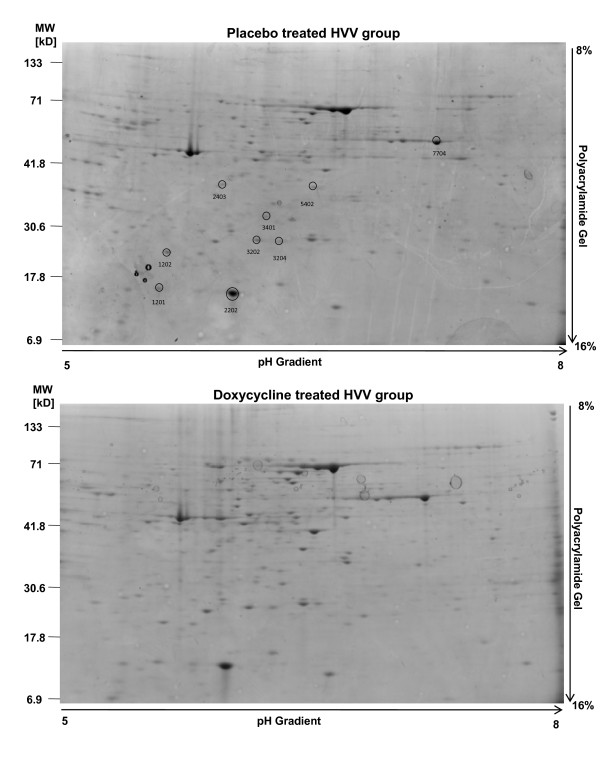
**Representative 2-DE gels of proteins from the treated with doxycycline group and from placebo group**. Circles represent affected protein spots while the numbers (beside the circles) identify the protein spots from quantitative analysis using PDQuest measurement software (BioRad).

**Figure 4:**
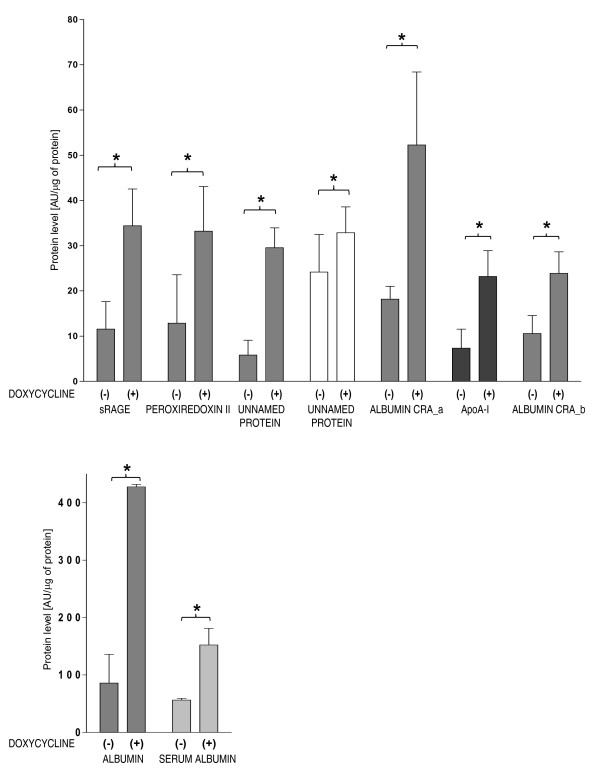
**Quantitative analysis of proteins for which levels significantly increased after treatment with doxycycline**. n = 6/group. *p < 0.05

Approximately 220 protein spots were detected in each gel (Figure 3). Using an arbitrary set sensitivity threshold, based on minimum peak value sensitivity and a noise filter level, only nine proteins have shown significant changes between analyzed groups (Figure 4). The results of densitometric analysis of protein spots from lungs subjected to ventilation after pretreatment with doxycycline as well as from placebo treated group are shown in Figure 4. The levels of all 9 proteins were significantly higher in the doxycycline treated HVV group as compared to the placebo treated HVV group. The results of protein identification by mass spectrometry (MS) analysis are presented in Table 1 and the structural information for the peptides used for protein identification is shown in Additional file 1 - Table S1. We have identified 9 proteins including: soluble receptor for advanced glycation endproduct (sRAGE), Apoliporotein A-I (ApoA-I), Peroxiredoxine II (Prx II) and four molecular forms of albumin. Also, two protein spots were identified as unnamed proteins.

**Table 1:** Results of identification of protein spots changed by doxycycline treatment

Protein Spot (SSP)	Protein Score	Protein Score C.I. %	Total Ion Score	Total Ion C.I. %	Protein Identification
1201	122	100	111	100	sRAGE
1202	621	100	537	100	Peroxiredoxin II
2202	436	100	400	100	Albumin, isoform CRA_b
2403	249	100	135	100	Unnamed protein
3202	511	100	452	100	Unnamed protein
3204	170	100	164	100	Albumin, isoform CRA_a
3401	1170	100	918	100	Apolipoprotein A-I
5402	883	100	701	100	Albumin
7704	1150	100	918	100	Serum Albumin

Next, the analysis of correlations between protein levels was performed in order to assess the relationships, which might reflect a common pathophysiological pathway leading to the changes in their levels. Positive, statistically significant correlations between levels of identified proteins were observed and are presented in Table 2.

**Table 2:** Correlations between levels of identified protein

Proteins	R=	p=
**sRAGE and Albumin**	0.87	0.001
**sRAGE and Albumin, isoform CRA_a**	0.96	0.0001
**sRAGE and Apolipotrotein A-I**	0.72	0.029
**Peroxiredoxin II and Serum albumin**	0.86	0.005
**Albumin and Albumin, isoform CRA_a**	0.85	0.002
**Albumin and Unnamed protein (SSP 2403)**	0.73	0.022
**Apolipoprotein A-I and Albumin, isoform CRA_a**	0.67	0.049

### Mechanical ventilation and MMP-9 activity

**Figure 5:**
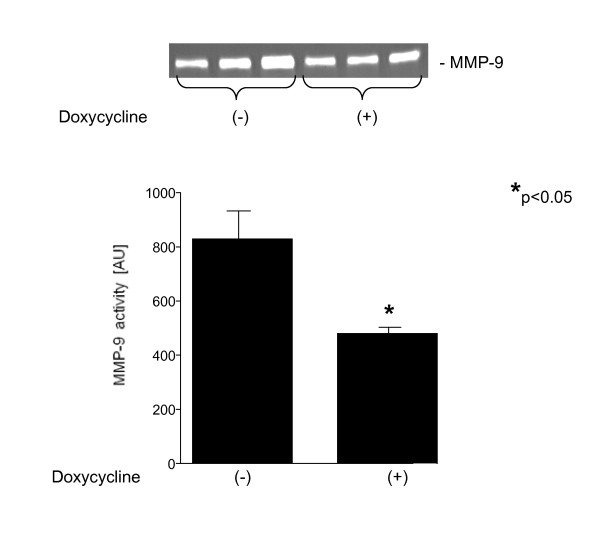
**Quantitative analysis of the MMP-9 activity with (+) and without (-) pretreatment with doxycycline**. Inset is a representative zymography gel. *p < 0.05

The activity of MMP-9 in lung homogenates in both groups is shown in Figure 5. Treatment with doxycycline decreased MMP-9 activity (475.2 ± 28.1 AU *vs. *813.8 ± 119.5 AU in placebo treated HVV group, respectively, p < 0.05).

### Immunoblot analysis of identified proteins

**Figure 6:**
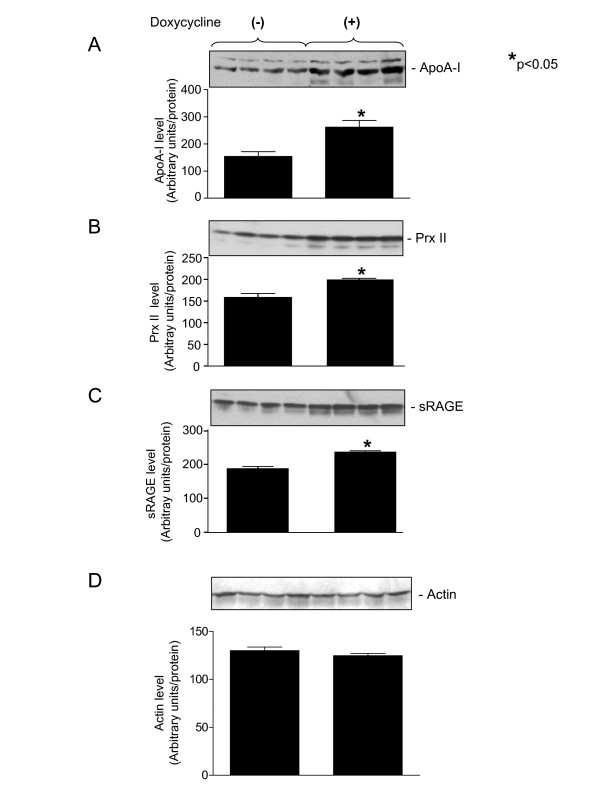
**Immunoblot analysis of proteins identified from 2-DE and mass spectrometry analysis**. Lung samples from the groups treated with doxycycline or with placebo were analyzed. A. ApoA-I -- apolipoprotein A-I, B. Prx II -- peroxiredoxin II, C. sRAGE -- soluble receptor for advanced glycation end-product. D. Loading control for immunoblots (Actin). *p < 0.05.

Immunoblot analysis for ApoA-I, Prx II and sRAGE has revealed significant increase in the levels of analyzed proteins in the group treated with doxycycline as compared to the placebo group (Figure 6). Loading control is shown in Figure 6D.

## Discussion

Proteins play an important role in regulation of biological systems; therefore, they are among the most common diagnostic and therapeutic targets in medicine. Hence, studying changes in the proteome should lead to the discovery of novel diagnostic markers and therapeutic strategies. Ventilation induced lung injury (VILI) has been shown to be associated with alteration of a broad variety of proteins within lungs (such as IL8, nuclear factor Nrf2, surfactant associated proteins A and D as well as pro-B cell enhancing factor (PBEF)) [28-31]. In this rat model of VILI, high tidal volume ventilation (HVV) should result in changes similar in nature but greater in magnitude as compared to those observed in mechanically ventilated patients in emergency units.

We have shown that the use of doxycycline effectively reduces changes in function of lungs ventilated mechanically with high tidal volume and decreases activity of MMP-9 within pulmonary tissue. Moreover, as we have demonstrated, treatment with doxycycline results in up-regulation of several proteins which could potentially explain observed minimization of the susceptibility of pulmonary tissue to VILI.

In this study, the proteins for which levels have significantly increased have been shown to play a protective role in lung injury under various pathological conditions. Therefore, treatment aimed at preventing their alteration, such as hereby demonstrated with doxycycline, could be considered as an important therapeutic aim in limitation of VILI. We have observed significant changes in concentrations of nine proteins identified by mass spectrometry (MS) analysis (soluble receptor for advanced glycation endproduct (sRAGE), apoliporotein A-I (ApoA-I), peroxiredoxin II (Prx II), four molecular forms of albumin and two unnamed proteins). Since the antibodies for unnamed proteins identified by MS analysis are commercially unavailable, and albumins constitute a heterogeneous group of proteins with diverse antigen specificity, we decided to analyze only three recognized proteins by immunoblot (ApoA-I, sRAGE and Prx II). Detected proteins were up-regulated in doxycycline treated group which is thus consistent with the 2-DE results.

Our study reveals an increase in ApoA-I level in pulmonary tissue after treatment with doxycycline. Since ApoA-I overexpression has been demonstrated to play a protective role in lipopolisacharide (LPS) induced systemic inflammation and multiple organ damage in mice [32], the increase in ApoA-I level, which we observed, can thus play an important role in limitation of VILI as well. ApoA-I could effectively protect against endotoxemia and acute lung damage. The potential mechanism might be related to inhibition of inflammatory cytokine release from macrophages.

The next altered protein which we detected in our experimental model of VILI is peroxiredoxin II (Prx II). Our results indicate that treatment with doxycycline prevents a decrease in levels of Prx II; therefore the antioxidative capacity of pulmonary tissue might be increased, which could result in lower susceptibility to ventilation induced damage. Various Prx enzymes were tested for their capacity to scavenge peroxynitrite. Some of these enzymes were reported to catalytically reduce peroxynitrite to nitrite rapidly enough to forestall the damage of cellular components [33]. Subsequently, different Prx enzymes have been shown to be associated with peroxynitrite reductase activity [34], mammalian Prx V [35], and Prx VI [36]. The change in Prx II level in response to the treatment with doxycycline, observed in this study, could be an important defense mechanism limiting peroxynitrite-dependent lung damage during mechanical ventilation.

Furthermore, this study demonstrates an increase in the expression of soluble receptor for advanced glycation endproduct (sRAGE) due to pretreatment with doxycycline. Recently, several carboxyl-terminal truncated isoforms of RAGE, such as soluble RAGE (sRAGE) and endogenous secretory RAGE (esRAGE) were identified in the lung of both humans and mice [37]. sRAGE which is up-regulated in the alveolar space in response to intratracheal LPS challenge, mitigates LPS-induced inflammatory events in the lung, including neutrophil infiltration, increased lung permeability, edema formation, production of inflammatory cytokines, and NF-kB. These findings indicate that RAGE plays a critical role in pathogenesis of lung injury and the blockade of RAGE signaling by sRAGE could be protective against the development of lung injury [38]. Moreover, sRAGE may be a useful biological marker of alveolar epithelial injury and impaired alveolar fluid clearance [39-45].

Two altered protein spots were identified as unnamed proteins (SSP 2403: MW≈38kD, pI≈5.9 and SSP 3202: MW≈28kD, pI≈6.2). The levels of both proteins were significantly higher after pretreatment with doxycycline. The exact role of these proteins in pulmonary pathology remains unknown and needs to be further investigated. In our study we also observed a decreased level of albumins (albumin isoform CRA_a [NCBI:EDL88549], albumin [NCBI:NP_599153], serum albumin [NCBI:P02770] and albumin isoform CRA_b [NCBI:EDL92173]) in the placebo treated HVV group which could reflect protein loss from the extracellular space within pulmonary tissue as well as transudate of the albumin-poor fluid into the extracellular space. The increase in albumin levels in pulmonary tissue after doxycycline treatment might be due the restoration of endothelial integrity in response to MMP-9 inhibition resulting in limitation of fluid transudation. However, further research is required in order to elucidate whether this phenomenon plays a role in pathogenesis of VILI or is only additional effect of treatment with doxycycline.

Although the identified proteins are involved in different regulatory mechanisms, there might be one common mechanism leading to their alteration during pathology, since positive correlations were observed. Since inhibition of MMP-9 by doxycycline prevents protein alteration during HVV, a degradation of these proteins by MMP-9 might be postulated. Hence, treatment with doxycycline could be an effective pharmacological intervention attenuating this phenomenon and resulting in decreased lung damage during mechanical ventilation. Nevertheless, further research is required in order to verify this hypothesis and to assess its relevance in clinical practice.

Two studies identified elevated MMP-9 levels in the lungs of newborns with acute respiratory distress syndrome [46,47]. Although these studies demonstrate that elevated MMP-9 levels distinguish individuals with acute lung disease from normal controls, it is not clear whether differences in MMP-9 levels/activity have pathophysiological or prognostic significance. In this study we have shown for the first time that the use of doxycycline in an animal model of a high volume VILI not only decreases the MMP-9 activity but also prevents alteration of nine proteins in pulmonary tissue. Thus, decreasing MMP-9 activity by use of an MMP-inhibitor may protect the lungs from VILI. However, the question of whether the changes which we observed in the pulmonary proteome in response to the treatment with doxycycline are directly due to MMP-9 inhibition or whether they are a result of other mechanisms of drug action remains unanswered.

## Conclusions

In conclusion, we found that treatment with doxycycline prevents alteration of proteins which are shown to play a protective role in lung injury. Moreover, we show positive correlations between their levels which might reflect a common pathway of their alteration that is effectively inhibited by use of doxycycline. Identified proteins could be a proteolytic target for MMP-9 and therefore the use of doxycycline as MMP-9 inhibitor could prevent their degradation during high tidal volume ventilation.

A better understanding of these pathologic mechanisms will help not only in alleviating the side effects of mechanical forces but also in the development of new therapeutic strategies. It is necessary to discover pharmacologic targets to modulate the molecular effects of lung stretch in order to minimize the negative consequences of mechanical ventilation. Moreover, an effective therapy for these disorders needs to be instituted during early stages of the disease, prior to the development of extensive lung destruction and fibrosis.

## Materials and methods

This study was carried out with the approval of the Animal Research Ethics Board of the University Committee on Animal Care and Supply (University of Saskatchewan) and conformed to the principles embodied in the Declaration of Helsinki.

### Experimental protocol

#### Model of ventilation-induced lung injury

We studied two groups of twenty adult Wistar rats (weight 357 ± 41 g) using a high volume ventilation protocol known to produce ventilation-induced lung injury (VILI) [48] (Figure 1). All animals were pretreated orally late in the day (at approximately 9 PM) prior to the procedure day. The first group of animals was given 2 mg/kg of lactose as placebo (placebo treated HVV group) and the second group was given 2 mg/kg of doxycycline hyclate (Doxycin, RIVA Labs., Blainville, PQ, Canada) (doxycycline treated HVV group). Next morning (at approximately 7 AM) anesthesia was induced by intraperitoneal injection of sodium pentobarbital (60 mg/kg) and was maintained by a continuous intravenous infusion of sodium pentobarbital (25 mg/kg/h). The trachea was then exposed and an endotracheal tube (13-15 gauges) inserted. The rat body temperature was monitored by a rectal probe and body temperature was maintained near 37°C by a heating pad. A small animal ventilator (Harvard rodent ventilator, Model 683, Harvard Apparatus, Holliston, Mass., USA) was connected to the steel tracheal tube and all animals initially received standardized ventilator settings (Tidal volume (Vt) = 7.5 ml/kg, rate: 60 breaths/min; FiO_2 _= 1.0). Pressure at the airway opening was measured using an air-phase transducer (P45, Validyne Eng. Corp., Northridge, CA) and continuously displayed. A catheter (25 gauges, Insyte IV catheter, Benton Dickson, Sandy, Utah, USA) was inserted into the carotid artery and systemic arterial blood pressure was monitored throughout (Space ALABS MODEL 510, Squibb, Hollsboro, OR). All animals received a continuous infusion of normal saline at 6 ml/kg/h as maintenance fluid. Two groups of animals received 20 min of high volume ventilation (HVV) (Vt = 30 ml/kg, rate = 15/min, PEEP = 8 cm H_2_O). After the 20 min test ventilation all groups were ventilated further for 240 min on the low volume regimen (Vt = 7.5 ml/kg, rate = 60/min). During periods of HVV, the respiratory rate was decreased in order to maintain constant minute ventilation and physiologic blood pH.

At the end of the ventilation period, 1 ml of arterial blood was collected for gas analysis using a heparinized syringe (CIBA-Corning 238 pH - Blood Gas Analyzer, Midfield, Mass.). The pulmonary compliance was measured using the method described by LeSouef *et al *[49]. Afterwards, the animals were euthanized and lungs were rapidly removed as a block. The lungs were rinsed/perfused for one minute with normal saline solution and snap-frozen in liquid nitrogen for subsequent biochemical analyses.

### Lung Tissue Assays

#### Preparation of lung extracts

Protein samples for 2-dimensional electrophoresis (2-DE) were prepared at room temperature by mixing frozen (-80°C), powdered tissue (40 to 60 mg wet weight) with 200 μL of rehydration buffer (8 mM urea, 4% CHAPS, 10 mM DTT, 0.2% Bio-Lytes 3/10 [BioRad]). Samples were sonicated twice for 5 s and centrifuged for 10 min at 10 000 *g *at 4°C to remove any insoluble particles. Protein content of the extract in rehydration buffer was measured with the BioRad protein assay. For other biochemical studies, frozen powdered tissue was homogenized on ice in 50 mM Tris-HCl (pH 7.4) containing 3.1 mM sucrose, 1 mM DTT, 10 μg/ml leupeptin, 10 μg/ml soybean trypsin inhibitor, 2 μg/ml aprotinin and 0.1% Triton X-100. Homogenates were centrifuged at 10 000 *g *at 4°C for 10 min and the supernatant was collected and stored at -80°C until further use.

#### Assessment of MMP-9 activity using zymography

Gelatin zymography was performed as described previously [50]. Briefly, lung extract preparations (60 μg of protein) were applied to 8% polyacrylamide gel copolymerized with 2 mg/ml gelatin. After electrophoresis, gels were rinsed 3 times for 20 min each in 2.5% Triton X-100 to remove SDS. The gels were then washed twice in incubation buffer (50 mM Tris-HCl, 5 mM CaCl_2_, 150 mM NaCl, and 0.05% NaN_3_) for 20 min each at room temperature and then incubated in incubation buffer at 37°C for 24 h. The gels were stained in 0.05% Coomassie Brilliant Blue G in a mixture of methanol: acetic acid: water (2.5:1:6.5, v:v) and destained in aqueous 4% methanol: 8% acetic acid (v:v). Developed gels were scanned with a GS-800 densitometer (BioRad) and the MMP-9 activity was measured using Quantity One measurement software 4.6 (BioRad).

### 2-dimensional gel electrophoresis (2-DE)

After preparing the tissue extract, a protein solution (0.4 mg) was used to rehydrate a dried first-dimension strip (IPG strip, pH 5-8, 11 cm, BioRad) for 18 h at 20°C under mineral oil. Next, the strips were subjected to isoelectrofocusing (IEF) using a BioRad Protean IEF cell apparatus (BioRad) with focusing parameters as follows: step 1: 15 min with end voltage at 250 V; step 2: 150 min with end voltage at 8000 V; step 3: 35000 V-hours (approximately 260 min). After IEF, the strips were reduced and alkylated in 1% DTT and 2.5% iodoacetamide (in ReadyPrep 2DE Starter Kit Equilibration Buffer I and II, respectively, BioRad). The proteins were separated by size in the second dimension by SDS-PAGE on 8-16% Criterion precast gels (BioRad). To minimize variations in resolving proteins during the 2-DE run, all gels were run simultaneously using a Criterion Dodeca Cell (BioRad). All the gels were stained in the same Coomassie Brilliant Blue R 250 (BioRad) staining bath as previously described [51].

Developed gels were scanned using a GS-800 calibrated densitometer (BioRad). The intensity of spots from 2-DE gels was measured using PDQuest 7.1 software (BioRad).

### Mass spectrometry (MS) Analysis

Selected protein spots were manually excised from the 2-DE gels. These spots were then processed using a MassPrep Station from Micromass using the methods supplied by the manufacturer. Briefly, the excised gel fragment containing the protein spot was first destained, reduced, alkylated, digested with trypsin and extracted. Mass analysis of the trypsin digest was performed on MALDI-TOF Voyager DE-Pro from Applied Biosystems. A mass deviation of 0.5 was tolerated and one missed cleavage site was allowed. Resulting values from mass spectrometry analysis for monoisotopic peaks were used to search against the NCBInr and Swiss-Prot databases with *Ratus norvegicus *specified. We used the Mascot http://www.matrixscience.com search engine to search the protein database for protein identification. The Mowse scoring algorithm [52] was used for justification of accuracy of protein identification and is incorporated in the Mascot search engine.

### Immunoblotting

We separated 30 μg of protein from each lung extract using Criterion pre-cast gels (8 to 16%) (BioRad) and transferred the protein to a polyvinylidene difluoride membrane (Bio-Rad). Apolipoprotein A-I (ApoA-I) was identified using a rabbit polyclonal anti-ApoA-I antibody (from Santa Cruz Biotechnology Inc.) at dilution 1:200 and a goat anti-rabbit IgG-HRP as secondary antibody (from Santa Cruz Biotechnology Inc.) at dilution 1:4000. Peroxiredoxin II (Prx II) was identified using a rabbit polyclonal anti-Prx II antibody (from Santa Cruz Biotechnology Inc.) at dilution 1:200 and goat anti-rabbit IgG-HRP as secondary antibody (from Santa Cruz Biotechnology Inc.) at dilution 1:2000. Soluble receptor for advanced glycosylation endproduct (sRAGE) was identified using a goat polyclonal anti-RAGE (N16) antibody against the N-terminus of RAGE (from Santa Cruz Biotechnology Inc.) at dilution 1:200 and donkey anti-goat IgG-HRP as secondary antibody (from Santa Cruz Biotechnology Inc.) at dilution 1:2000. Actin as a loading control was assessed with a mouse anti-actin antibody (Millipore) at dilution 1:150 and goat anti-mouse IgG-HRP (BioRad) as secondary antibody at dilution 1:1000. Bands detected by chemiluminescence were scanned using VersaDoc Gel Imaging System (BioRad) and measured by Quantity One software 4.6 (BioRad).

### Statistical analysis

The protein spot levels as well as MMP-9 activity were evaluated by Mann-Whitney U-test and student's t-test. Functional data was analysed using Kruskal-Wallis test followed by Dunns test for pairs. Correlations between the levels of proteins were assessed by Spearman Moment. Data are expressed as mean ± SEM. Differences were considered significant at p < 0.05.

## Competing interests

The authors declare that they have no competing interests.

## Authors' contributions

AD contributed to experimental part, data analysis and manuscript preparation. TSH contributed to experimental design, data analysis animal model experiments and manuscript preparation. DP contributed to experimental part, data analysis and manuscript preparation. JS and JFB contributed to experimental part. DHJ contributed to study design. GS was responsible for supervision and preparation of the manuscript. All authors read and approved the final manuscript.

## Supplementary Material

Additional file 1 **Table S1**. Structural information of the peptides used for protein identification Click here for file
