# Supplementary material for: Effects of MMP-9 inhibition by doxycycline on proteome of lungs in high tidal volume mechanical ventilation-induced acute lung injury
Source: Proteome Sci. 2010 Jan 29;8:3. doi: 10.1186/1477-5956-8-3 (PMC2824689; doi:10.1186/1477-5956-8-3)
Supplement: Additional file 1 — Table S1. Structural information of the peptides used for protein identification [file 1477-5956-8-3-S1.PPT]

## Slide 1
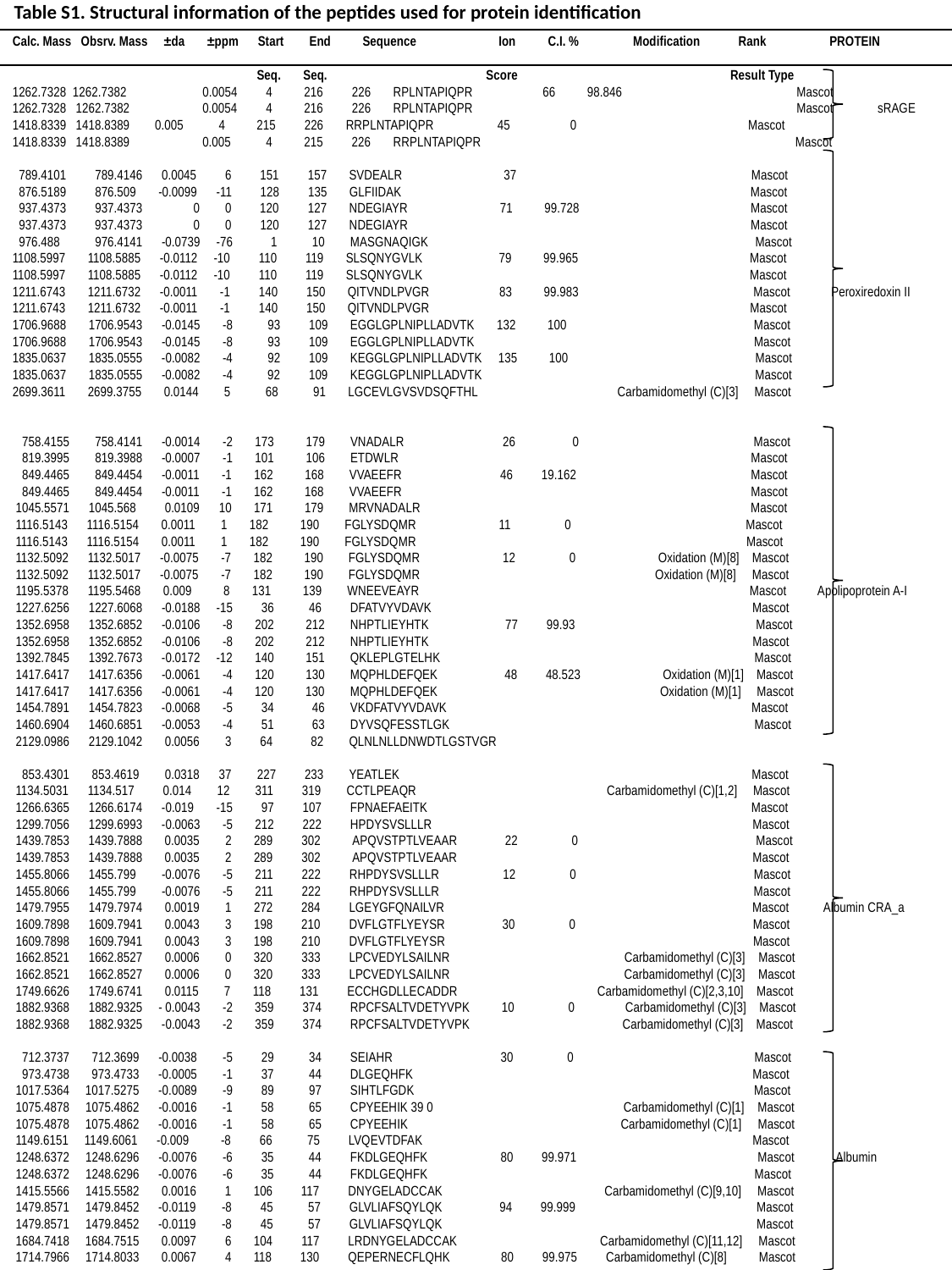

Table S1. Structural information of the peptides used for protein identification
Calc. Mass Obsrv. Mass ±da ±ppm Start End Sequence Ion C.I. % Modification Rank PROTEIN
 Seq. Seq. Score Result Type
1262.7328 1262.7382	 0.0054 4 216 226 RPLNTAPIQPR 66 98.846 Mascot
1262.7328 1262.7382 	 0.0054 4 216 226 RPLNTAPIQPR Mascot sRAGE
1418.8339 1418.8389 0.005 4 215 226 RRPLNTAPIQPR 45 0 Mascot
1418.8339 1418.8389 	 0.005 4 215 226 RRPLNTAPIQPR Mascot
 789.4101 789.4146 0.0045 6 151 157 SVDEALR 37 Mascot
 876.5189 876.509 -0.0099 -11 128 135 GLFIIDAK Mascot
 937.4373 937.4373 0 0 120 127 NDEGIAYR 71 99.728 Mascot
 937.4373 937.4373 0 0 120 127 NDEGIAYR Mascot
 976.488 976.4141 -0.0739 -76 1 10 MASGNAQIGK Mascot
1108.5997 1108.5885 -0.0112 -10 110 119 SLSQNYGVLK 79 99.965 Mascot
1108.5997 1108.5885 -0.0112 -10 110 119 SLSQNYGVLK Mascot
1211.6743 1211.6732 -0.0011 -1 140 150 QITVNDLPVGR 83 99.983 Mascot Peroxiredoxin II
1211.6743 1211.6732 -0.0011 -1 140 150 QITVNDLPVGR Mascot
1706.9688 1706.9543 -0.0145 -8 93 109 EGGLGPLNIPLLADVTK 132 100 Mascot
1706.9688 1706.9543 -0.0145 -8 93 109 EGGLGPLNIPLLADVTK Mascot
1835.0637 1835.0555 -0.0082 -4 92 109 KEGGLGPLNIPLLADVTK 135 100 Mascot
1835.0637 1835.0555 -0.0082 -4 92 109 KEGGLGPLNIPLLADVTK Mascot
2699.3611 2699.3755 0.0144 5 68 91 LGCEVLGVSVDSQFTHL Carbamidomethyl (C)[3] Mascot
 758.4155 758.4141 -0.0014 -2 173 179 VNADALR 26 0 Mascot
 819.3995 819.3988 -0.0007 -1 101 106 ETDWLR Mascot
 849.4465 849.4454 -0.0011 -1 162 168 VVAEEFR 46 19.162 Mascot
 849.4465 849.4454 -0.0011 -1 162 168 VVAEEFR Mascot
 1045.5571 1045.568 0.0109 10 171 179 MRVNADALR Mascot
 1116.5143 1116.5154 0.0011 1 182 190 FGLYSDQMR 11 0 Mascot
 1116.5143 1116.5154 0.0011 1 182 190 FGLYSDQMR Mascot
 1132.5092 1132.5017 -0.0075 -7 182 190 FGLYSDQMR 12 0 Oxidation (M)[8] Mascot
 1132.5092 1132.5017 -0.0075 -7 182 190 FGLYSDQMR Oxidation (M)[8] Mascot
 1195.5378 1195.5468 0.009 8 131 139 WNEEVEAYR Mascot Apolipoprotein A-I
 1227.6256 1227.6068 -0.0188 -15 36 46 DFATVYVDAVK Mascot
 1352.6958 1352.6852 -0.0106 -8 202 212 NHPTLIEYHTK 77 99.93 Mascot
 1352.6958 1352.6852 -0.0106 -8 202 212 NHPTLIEYHTK Mascot
 1392.7845 1392.7673 -0.0172 -12 140 151 QKLEPLGTELHK Mascot
 1417.6417 1417.6356 -0.0061 -4 120 130 MQPHLDEFQEK 48 48.523 Oxidation (M)[1] Mascot
 1417.6417 1417.6356 -0.0061 -4 120 130 MQPHLDEFQEK Oxidation (M)[1] Mascot
 1454.7891 1454.7823 -0.0068 -5 34 46 VKDFATVYVDAVK Mascot
 1460.6904 1460.6851 -0.0053 -4 51 63 DYVSQFESSTLGK Mascot
 2129.0986 2129.1042 0.0056 3 64 82 QLNLNLLDNWDTLGSTVGR
 853.4301 853.4619 0.0318 37 227 233 YEATLEK Mascot
 1134.5031 1134.517 0.014 12 311 319 CCTLPEAQR Carbamidomethyl (C)[1,2] Mascot
 1266.6365 1266.6174 -0.019 -15 97 107 FPNAEFAEITK Mascot
 1299.7056 1299.6993 -0.0063 -5 212 222 HPDYSVSLLLR Mascot
 1439.7853 1439.7888 0.0035 2 289 302 APQVSTPTLVEAAR 22 0 Mascot
 1439.7853 1439.7888 0.0035 2 289 302 APQVSTPTLVEAAR Mascot
 1455.8066 1455.799 -0.0076 -5 211 222 RHPDYSVSLLLR 12 0 Mascot
 1455.8066 1455.799 -0.0076 -5 211 222 RHPDYSVSLLLR Mascot
 1479.7955 1479.7974 0.0019 1 272 284 LGEYGFQNAILVR Mascot Albumin CRA_a
 1609.7898 1609.7941 0.0043 3 198 210 DVFLGTFLYEYSR 30 0 Mascot
 1609.7898 1609.7941 0.0043 3 198 210 DVFLGTFLYEYSR Mascot
 1662.8521 1662.8527 0.0006 0 320 333 LPCVEDYLSAILNR Carbamidomethyl (C)[3] Mascot
 1662.8521 1662.8527 0.0006 0 320 333 LPCVEDYLSAILNR Carbamidomethyl (C)[3] Mascot
 1749.6626 1749.6741 0.0115 7 118 131 ECCHGDLLECADDR Carbamidomethyl (C)[2,3,10] Mascot
 1882.9368 1882.9325 - 0.0043 -2 359 374 RPCFSALTVDETYVPK 10 0 Carbamidomethyl (C)[3] Mascot
 1882.9368 1882.9325 -0.0043 -2 359 374 RPCFSALTVDETYVPK Carbamidomethyl (C)[3] Mascot
 712.3737 712.3699 -0.0038 -5 29 34 SEIAHR 30 0 Mascot
 973.4738 973.4733 -0.0005 -1 37 44 DLGEQHFK Mascot
 1017.5364 1017.5275 -0.0089 -9 89 97 SIHTLFGDK Mascot
 1075.4878 1075.4862 -0.0016 -1 58 65 CPYEEHIK 39 0 Carbamidomethyl (C)[1] Mascot
 1075.4878 1075.4862 -0.0016 -1 58 65 CPYEEHIK Carbamidomethyl (C)[1] Mascot
 1149.6151 1149.6061 -0.009 -8 66 75 LVQEVTDFAK Mascot
 1248.6372 1248.6296 -0.0076 -6 35 44 FKDLGEQHFK 80 99.971 Mascot Albumin
 1248.6372 1248.6296 -0.0076 -6 35 44 FKDLGEQHFK Mascot
 1415.5566 1415.5582 0.0016 1 106 117 DNYGELADCCAK Carbamidomethyl (C)[9,10] Mascot
 1479.8571 1479.8452 -0.0119 -8 45 57 GLVLIAFSQYLQK 94 99.999 Mascot
 1479.8571 1479.8452 -0.0119 -8 45 57 GLVLIAFSQYLQK Mascot
 1684.7418 1684.7515 0.0097 6 104 117 LRDNYGELADCCAK Carbamidomethyl (C)[11,12] Mascot
 1714.7966 1714.8033 0.0067 4 118 130 QEPERNECFLQHK 80 99.975 Carbamidomethyl (C)[8] Mascot

## Slide 2
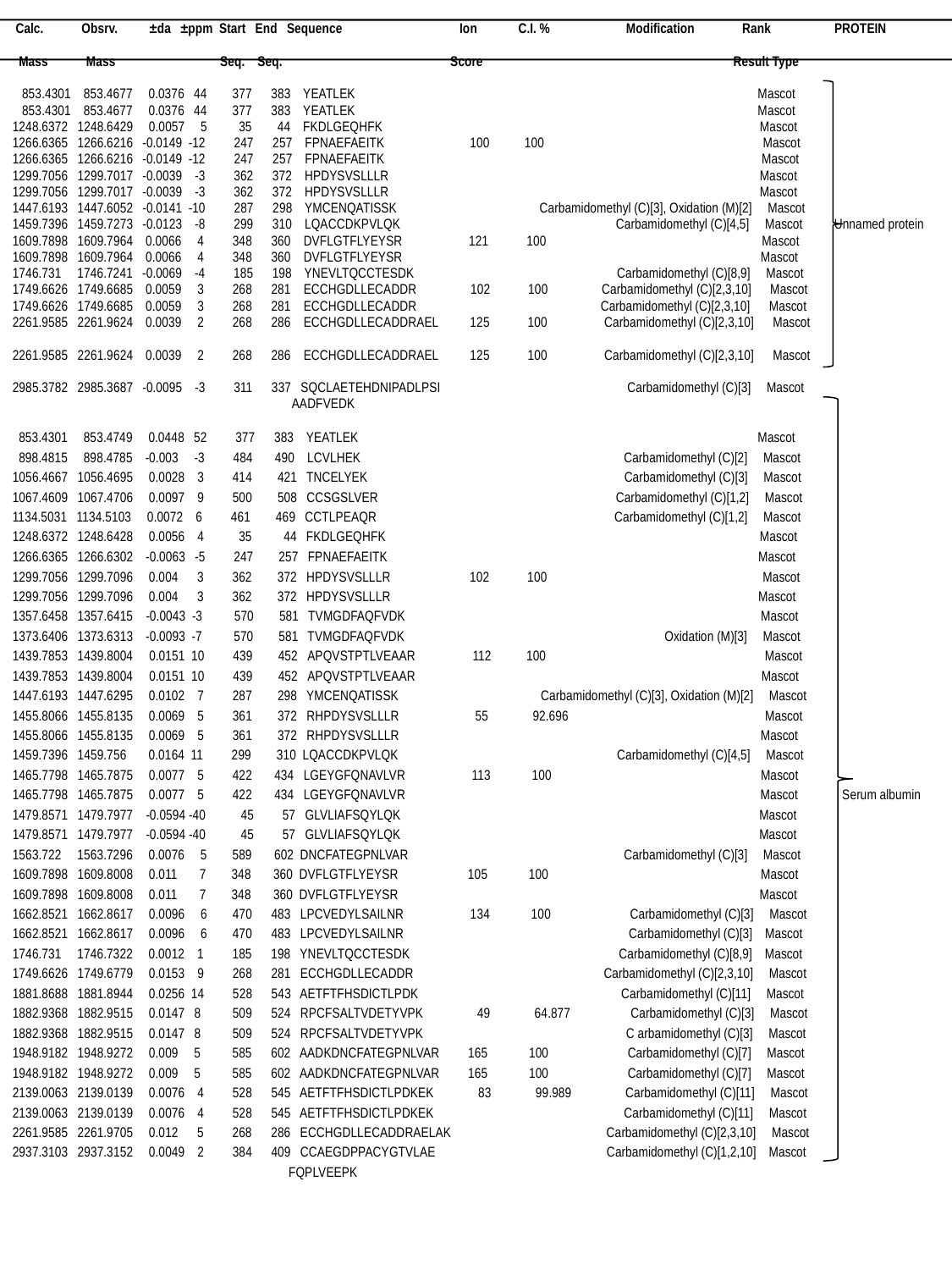

Calc. Obsrv. ±da ±ppm Start End Sequence Ion C.I. % Modification Rank PROTEIN
 Mass Mass Seq. Seq. Score Result Type
 853.4301 853.4677 0.0376 44 377 383 YEATLEK Mascot
 853.4301 853.4677 0.0376 44 377 383 YEATLEK Mascot
1248.6372 1248.6429 0.0057 5 35 44 FKDLGEQHFK Mascot
1266.6365 1266.6216 -0.0149 -12 247 257 FPNAEFAEITK 100 100 Mascot
1266.6365 1266.6216 -0.0149 -12 247 257 FPNAEFAEITK Mascot
1299.7056 1299.7017 -0.0039 -3 362 372 HPDYSVSLLLR Mascot
1299.7056 1299.7017 -0.0039 -3 362 372 HPDYSVSLLLR Mascot
1447.6193 1447.6052 -0.0141 -10 287 298 YMCENQATISSK Carbamidomethyl (C)[3], Oxidation (M)[2] Mascot
1459.7396 1459.7273 -0.0123 -8 299 310 LQACCDKPVLQK Carbamidomethyl (C)[4,5] Mascot Unnamed protein
1609.7898 1609.7964 0.0066 4 348 360 DVFLGTFLYEYSR 121 100 Mascot
1609.7898 1609.7964 0.0066 4 348 360 DVFLGTFLYEYSR Mascot
1746.731 1746.7241 -0.0069 -4 185 198 YNEVLTQCCTESDK Carbamidomethyl (C)[8,9] Mascot
1749.6626 1749.6685 0.0059 3 268 281 ECCHGDLLECADDR 102 100 Carbamidomethyl (C)[2,3,10] Mascot
1749.6626 1749.6685 0.0059 3 268 281 ECCHGDLLECADDR Carbamidomethyl (C)[2,3,10] Mascot
2261.9585 2261.9624 0.0039 2 268 286 ECCHGDLLECADDRAEL 125 100 Carbamidomethyl (C)[2,3,10] Mascot
2261.9585 2261.9624 0.0039 2 268 286 ECCHGDLLECADDRAEL 125 100 Carbamidomethyl (C)[2,3,10] Mascot
2985.3782 2985.3687 -0.0095 -3 311 337 SQCLAETEHDNIPADLPSI Carbamidomethyl (C)[3] Mascot
 AADFVEDK
 853.4301 853.4749 0.0448 52 377 383 YEATLEK Mascot
 898.4815 898.4785 -0.003 -3 484 490 LCVLHEK Carbamidomethyl (C)[2] Mascot
1056.4667 1056.4695 0.0028 3 414 421 TNCELYEK Carbamidomethyl (C)[3] Mascot
1067.4609 1067.4706 0.0097 9 500 508 CCSGSLVER Carbamidomethyl (C)[1,2] Mascot
1134.5031 1134.5103 0.0072 6 461 469 CCTLPEAQR Carbamidomethyl (C)[1,2] Mascot
1248.6372 1248.6428 0.0056 4 35 44 FKDLGEQHFK Mascot
1266.6365 1266.6302 -0.0063 -5 247 257 FPNAEFAEITK Mascot
1299.7056 1299.7096 0.004 3 362 372 HPDYSVSLLLR 102 100 Mascot
1299.7056 1299.7096 0.004 3 362 372 HPDYSVSLLLR Mascot
1357.6458 1357.6415 -0.0043 -3 570 581 TVMGDFAQFVDK Mascot
1373.6406 1373.6313 -0.0093 -7 570 581 TVMGDFAQFVDK Oxidation (M)[3] Mascot
1439.7853 1439.8004 0.0151 10 439 452 APQVSTPTLVEAAR 112 100 Mascot
1439.7853 1439.8004 0.0151 10 439 452 APQVSTPTLVEAAR Mascot
1447.6193 1447.6295 0.0102 7 287 298 YMCENQATISSK Carbamidomethyl (C)[3], Oxidation (M)[2] Mascot
1455.8066 1455.8135 0.0069 5 361 372 RHPDYSVSLLLR 55 92.696 Mascot
1455.8066 1455.8135 0.0069 5 361 372 RHPDYSVSLLLR Mascot
1459.7396 1459.756 0.0164 11 299 310 LQACCDKPVLQK Carbamidomethyl (C)[4,5] Mascot
1465.7798 1465.7875 0.0077 5 422 434 LGEYGFQNAVLVR 113 100 Mascot
1465.7798 1465.7875 0.0077 5 422 434 LGEYGFQNAVLVR Mascot Serum albumin
1479.8571 1479.7977 -0.0594 -40 45 57 GLVLIAFSQYLQK Mascot
1479.8571 1479.7977 -0.0594 -40 45 57 GLVLIAFSQYLQK Mascot
1563.722 1563.7296 0.0076 5 589 602 DNCFATEGPNLVAR Carbamidomethyl (C)[3] Mascot
1609.7898 1609.8008 0.011 7 348 360 DVFLGTFLYEYSR 105 100 Mascot
1609.7898 1609.8008 0.011 7 348 360 DVFLGTFLYEYSR Mascot
1662.8521 1662.8617 0.0096 6 470 483 LPCVEDYLSAILNR 134 100 Carbamidomethyl (C)[3] Mascot
1662.8521 1662.8617 0.0096 6 470 483 LPCVEDYLSAILNR Carbamidomethyl (C)[3] Mascot
1746.731 1746.7322 0.0012 1 185 198 YNEVLTQCCTESDK Carbamidomethyl (C)[8,9] Mascot
1749.6626 1749.6779 0.0153 9 268 281 ECCHGDLLECADDR Carbamidomethyl (C)[2,3,10] Mascot
1881.8688 1881.8944 0.0256 14 528 543 AETFTFHSDICTLPDK Carbamidomethyl (C)[11] Mascot
1882.9368 1882.9515 0.0147 8 509 524 RPCFSALTVDETYVPK 49 64.877 Carbamidomethyl (C)[3] Mascot
1882.9368 1882.9515 0.0147 8 509 524 RPCFSALTVDETYVPK C arbamidomethyl (C)[3] Mascot
1948.9182 1948.9272 0.009 5 585 602 AADKDNCFATEGPNLVAR 165 100 Carbamidomethyl (C)[7] Mascot
1948.9182 1948.9272 0.009 5 585 602 AADKDNCFATEGPNLVAR 165 100 Carbamidomethyl (C)[7] Mascot
2139.0063 2139.0139 0.0076 4 528 545 AETFTFHSDICTLPDKEK 83 99.989 Carbamidomethyl (C)[11] Mascot
2139.0063 2139.0139 0.0076 4 528 545 AETFTFHSDICTLPDKEK Carbamidomethyl (C)[11] Mascot
2261.9585 2261.9705 0.012 5 268 286 ECCHGDLLECADDRAELAK Carbamidomethyl (C)[2,3,10] Mascot
2937.3103 2937.3152 0.0049 2 384 409 CCAEGDPPACYGTVLAE Carbamidomethyl (C)[1,2,10] Mascot
 FQPLVEEPK

## Slide 3
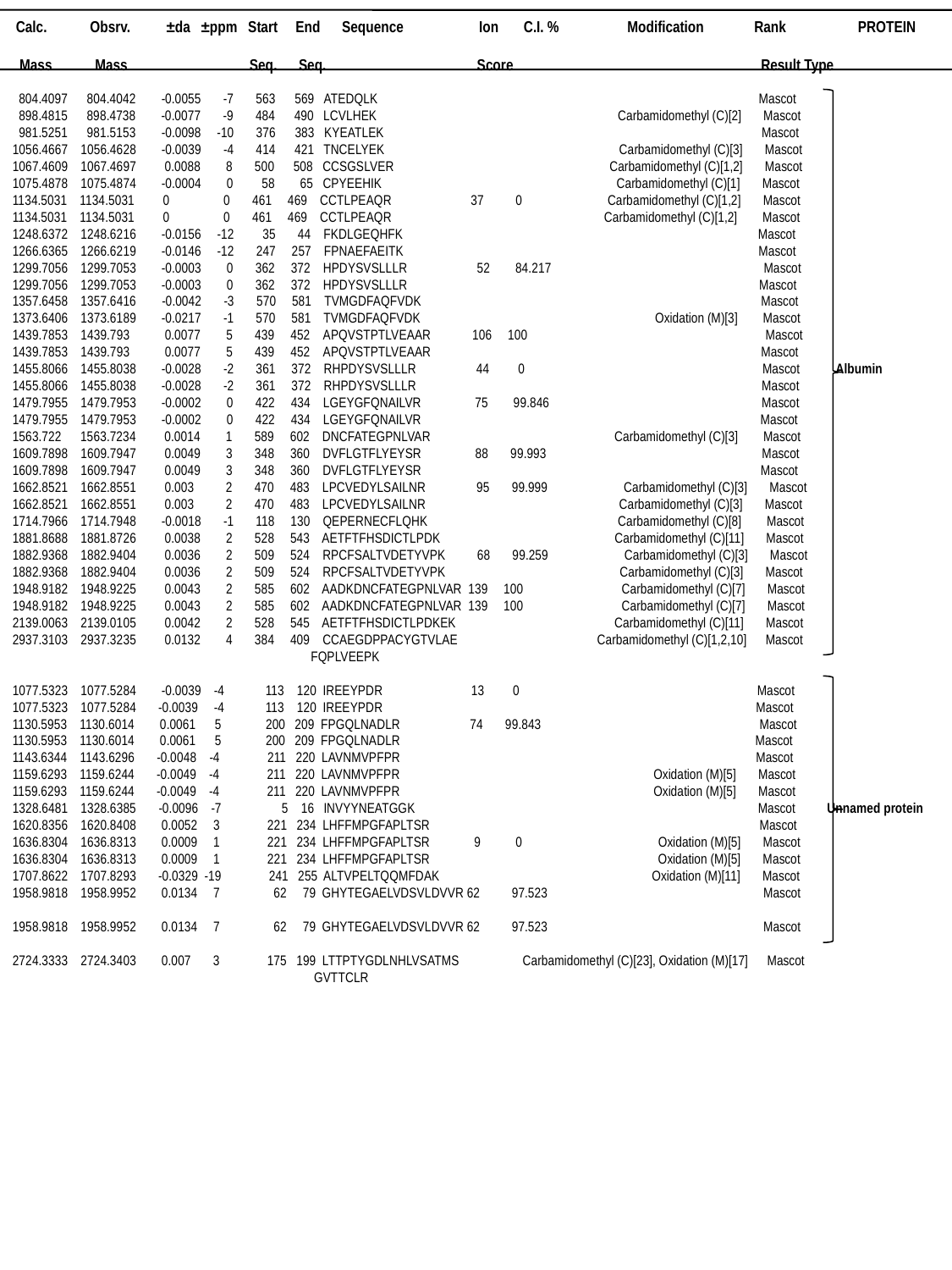

Calc. Obsrv. ±da ±ppm Start End Sequence Ion C.I. % Modification Rank PROTEIN
 Mass Mass Seq. Seq. Score Result Type
 804.4097 804.4042 -0.0055 -7 563 569 ATEDQLK Mascot
 898.4815 898.4738 -0.0077 -9 484 490 LCVLHEK Carbamidomethyl (C)[2] Mascot
 981.5251 981.5153 -0.0098 -10 376 383 KYEATLEK Mascot
1056.4667 1056.4628 -0.0039 -4 414 421 TNCELYEK Carbamidomethyl (C)[3] Mascot
1067.4609 1067.4697 0.0088 8 500 508 CCSGSLVER Carbamidomethyl (C)[1,2] Mascot
1075.4878 1075.4874 -0.0004 0 58 65 CPYEEHIK Carbamidomethyl (C)[1] Mascot
1134.5031 1134.5031 0 0 461 469 CCTLPEAQR 37 0 Carbamidomethyl (C)[1,2] Mascot
1134.5031 1134.5031 0 0 461 469 CCTLPEAQR Carbamidomethyl (C)[1,2] Mascot
1248.6372 1248.6216 -0.0156 -12 35 44 FKDLGEQHFK Mascot
1266.6365 1266.6219 -0.0146 -12 247 257 FPNAEFAEITK Mascot
1299.7056 1299.7053 -0.0003 0 362 372 HPDYSVSLLLR 52 84.217 Mascot
1299.7056 1299.7053 -0.0003 0 362 372 HPDYSVSLLLR Mascot
1357.6458 1357.6416 -0.0042 -3 570 581 TVMGDFAQFVDK Mascot
1373.6406 1373.6189 -0.0217 -1 570 581 TVMGDFAQFVDK Oxidation (M)[3] Mascot
1439.7853 1439.793 0.0077 5 439 452 APQVSTPTLVEAAR 106 100 Mascot
1439.7853 1439.793 0.0077 5 439 452 APQVSTPTLVEAAR Mascot
1455.8066 1455.8038 -0.0028 -2 361 372 RHPDYSVSLLLR 44 0 Mascot Albumin
1455.8066 1455.8038 -0.0028 -2 361 372 RHPDYSVSLLLR Mascot
1479.7955 1479.7953 -0.0002 0 422 434 LGEYGFQNAILVR 75 99.846 Mascot
1479.7955 1479.7953 -0.0002 0 422 434 LGEYGFQNAILVR Mascot
1563.722 1563.7234 0.0014 1 589 602 DNCFATEGPNLVAR Carbamidomethyl (C)[3] Mascot
1609.7898 1609.7947 0.0049 3 348 360 DVFLGTFLYEYSR 88 99.993 Mascot
1609.7898 1609.7947 0.0049 3 348 360 DVFLGTFLYEYSR Mascot
1662.8521 1662.8551 0.003 2 470 483 LPCVEDYLSAILNR 95 99.999 Carbamidomethyl (C)[3] Mascot
1662.8521 1662.8551 0.003 2 470 483 LPCVEDYLSAILNR Carbamidomethyl (C)[3] Mascot
1714.7966 1714.7948 -0.0018 -1 118 130 QEPERNECFLQHK Carbamidomethyl (C)[8] Mascot
1881.8688 1881.8726 0.0038 2 528 543 AETFTFHSDICTLPDK Carbamidomethyl (C)[11] Mascot
1882.9368 1882.9404 0.0036 2 509 524 RPCFSALTVDETYVPK 68 99.259 Carbamidomethyl (C)[3] Mascot
1882.9368 1882.9404 0.0036 2 509 524 RPCFSALTVDETYVPK Carbamidomethyl (C)[3] Mascot
1948.9182 1948.9225 0.0043 2 585 602 AADKDNCFATEGPNLVAR 139 100 Carbamidomethyl (C)[7] Mascot
1948.9182 1948.9225 0.0043 2 585 602 AADKDNCFATEGPNLVAR 139 100 Carbamidomethyl (C)[7] Mascot
2139.0063 2139.0105 0.0042 2 528 545 AETFTFHSDICTLPDKEK Carbamidomethyl (C)[11] Mascot
2937.3103 2937.3235 0.0132 4 384 409 CCAEGDPPACYGTVLAE Carbamidomethyl (C)[1,2,10] Mascot
 FQPLVEEPK
1077.5323 1077.5284 -0.0039 -4 113 120 IREEYPDR 13 0 Mascot
1077.5323 1077.5284 -0.0039 -4 113 120 IREEYPDR Mascot
1130.5953 1130.6014 0.0061 5 200 209 FPGQLNADLR 74 99.843 Mascot
1130.5953 1130.6014 0.0061 5 200 209 FPGQLNADLR Mascot
1143.6344 1143.6296 -0.0048 -4 211 220 LAVNMVPFPR Mascot
1159.6293 1159.6244 -0.0049 -4 211 220 LAVNMVPFPR Oxidation (M)[5] Mascot
1159.6293 1159.6244 -0.0049 -4 211 220 LAVNMVPFPR Oxidation (M)[5] Mascot
1328.6481 1328.6385 -0.0096 -7 5 16 INVYYNEATGGK Mascot Unnamed protein
1620.8356 1620.8408 0.0052 3 221 234 LHFFMPGFAPLTSR Mascot
1636.8304 1636.8313 0.0009 1 221 234 LHFFMPGFAPLTSR 9 0 Oxidation (M)[5] Mascot
1636.8304 1636.8313 0.0009 1 221 234 LHFFMPGFAPLTSR Oxidation (M)[5] Mascot
1707.8622 1707.8293 -0.0329 -19 241 255 ALTVPELTQQMFDAK Oxidation (M)[11] Mascot
1958.9818 1958.9952 0.0134 7 62 79 GHYTEGAELVDSVLDVVR 62 97.523 Mascot
1958.9818 1958.9952 0.0134 7 62 79 GHYTEGAELVDSVLDVVR 62 97.523 Mascot
2724.3333 2724.3403 0.007 3 175 199 LTTPTYGDLNHLVSATMS Carbamidomethyl (C)[23], Oxidation (M)[17] Mascot
 GVTTCLR
